# Atomic Phase Conjugation From a Bose Condensate

**DOI:** 10.6028/jres.101.058

**Published:** 1996

**Authors:** Elena V. Goldstein, Katja Plättner, Pierre Meystre

**Affiliations:** Optical Sciences Center and Department of Physics, University of Arizona, Tucson, AZ 85721

**Keywords:** Bose-Einstein condensate, dipole-dipole interaction, matter waves, Monte Carlo simulations, nonlinear atom optics, nonlinear master equation

## Abstract

We discuss the possibility of observing atomic phase conjugation from Bose condensates, and using it as a diagnostic tool to access the spatial coherence properties and to measure the lifetime of the condensate. We argue that since phase conjugation results from the scattering of a partial matter wave off the spatial grating produced by two other waves, it offers a natural way to directly measure such properties, and as such provides an attractive alternative to the optical methods proposed in the past.

## 1. Introduction

The study of many-body properties of ultra-cold and low density atomic samples is a topic of considerable experimental and theoretical interest. Substantial advances in this new subfield of atomic, molecular, and optical physics have been reported in the recent past, the most spectacular one being without doubt the observation of Bose-Einstein condensation in weakly interacting alkali vapors [1–3]. On the theoretical front, quantum field theories of the interaction between atoms and light fields are being developed [4–6], and are finding applications in the analysis of detection schemes for Bose condensates, nonlinear atom optics, etc.

While in the experiments reported so far, Bose-Einstein condensation has been detected via the velocity distribution of the atoms in the condensate, it is of interest to examine alternate techniques, such as optical methods, which could give clear and unambiguous signatures of the distinctions between a condensate and an incoherent atomic sample of the same density [7–12]. In this paper, we use the analogy between nonlinear optics and nonlinear atom optics to propose and analyze another measurement scheme sensitive to the coherence properties of the atomic sample under consideration. Based on this analogy, we have predicted in earlier work [13] that it should be possible to achieve atomic phase conjugation from an atomic condensate. Since phase conjugation is a *coherent* optical effect, which relies on the mutual coherence between the optical fields involved, we argue and demonstrate numerically that atomic phase conjugation is rapidly destroyed by incoherent processes such as spontaneous emission. Hence, it is not expected to occur for an incoherent atomic sample, and is a sensitive probe of the coherence properties and of the lifetime of the condensate.

Section 2 discusses our physical model, and proposes an experimental scheme where a Bose condensate is diffracted from an optical grating for atoms to produce two counter propagating condensate components, as well as a weak “probe” which is scattered off the condensate. In a manner reminiscent of the optical case, this configuration can lead to matter-wave phase conjugation via four-wave mixing. The basic approximations underlying our analysis, as well as the nonlinear master equation which models our system, are presented.

Section 3 reviews the coherent dynamics of the atoms and demonstrates that indeed, phase conjugation is possible in principle. Because the atoms under consideration are modeled as two-level systems, though, the theory of phase conjugation is somewhat more complicated than in the situation of degenerate four-wave mixing familiar in nonlinear optics, and is more reminiscent of the case where the polarization of the optical fields must be taken into account. Spontaneous emission is included in Sec. 4. In this case, the nonlinear Schrödinger equation used to describe the coherent effects is replaced by a nonlinear Hartree-Fock master equation, which is solved numerically. Spontaneous emission is shown to drastically reduce the amount of phase conjugation available, thereby demonstrating its sensitivity to the coherence of the condensate. In addition, we compare these results to the solution of a Hartree nonlinear Monte Carlo wave functions numerical scheme that we have recently proposed, thereby gaining useful intuition about the way a condensate’s description departs from Hartree as dissipation is increased. Finally, Sec. 5 is a summary and conclusion. Calculational details are relegated to appendices.

In order to avoid any confusion, we note now that the nonlinear Schrödinger equation that we use in this paper is *not* the Gross-Pitaevskii nonlinear Schrödinger equation familiar in the description of Bose condensates, but rather the nonlinear Schrödinger equation of atom optics [4,14]. In the former equation, the effective nonlinearity results from short-range interactions between ground state atoms, while in the latter case it results from the long-range, near-resonant dipole-dipole interaction between ground and excited atoms. Because the diffraction grating involved in our scheme induces real transitions between ground and excited atoms, this latter interaction is dominant, and hence we neglect the short range ground-ground interactions here. After having neglected this interaction, we do treat the dipole-dipole interaction as local, so that our nonlinear Schrödinger equation is itself local, just like the Gross-Pitaevskii equation. However, the physical situation it describes is quite different, and indeed, its Schrödinger field is vectorial rather than scalar.

## 2. Physical Model

**Fig. 1:**
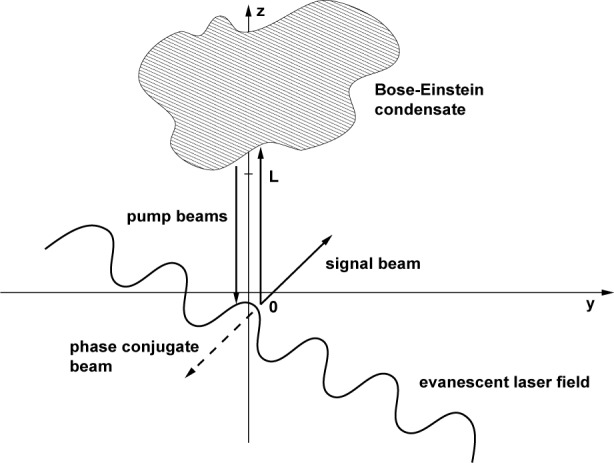
Configuration of the atomic beams incident on and diffracted from the evanescent light grating.

In analogy to the optical case of phase conjugation by four-wave mixing, we aim to realize a situation where a weak probe wave interacts nonlinearly with a strong “pump” wave to establish a grating from which a counter propagating pump wave is scattered to produce a phase-conjugate wave. A possible way to establish this geometry is shown in Fig. 1, which illustrates how a Bose condensate is diffracted by a matter waves grating produced by a periodic evanescent electromagnetic wave.

If the condensate impinges on the grating at the Bragg (Littrow) angle, the incident beam will be back-diffracted into two components, one propagating in the direction opposite to the incident wave (first-order diffraction) and the other, resulting from zeroth-order diffraction, at some angle from these “beams.” We already note at this point several important differences between this situation and the optical case: First, the waves are now de Broglie matter waves, instead of electromagnetic fields; second, these waves have several components, corresponding to all electronic states of relevance for the system at hand, so that the fields under consideration are vector fields; finally, due to two-body interactions between the atoms, their evolution is intrinsically nonlinear, so that no additional nonlinear “crystal” is required.

In general, atoms impinging on the grating in their ground electronic state will be diffracted in a superposition of ground and excited states, with two major consequences: the spontaneous decay of the excited atoms is an incoherent process that destroys the coherence between the various interacting waves, a mechanism that is expected to reduce the amount of phase conjugation achievable. In addition, a spontaneously emitted photon can be reabsorbed by another atom in the sample. This is the physical mechanism leading to the near-resonant dipole-dipole interaction, which is the major source of nonlinearity in the gedanken experiment at hand.

We have mentioned in the Introduction that the dynamics of a Bose condensate is described by the Gross-Pitaevskii nonlinear Schrödinger equation [15], in which the nonlinearity results from short-range two-body collisions with a scattering length of the order of the Bohr radius. In the presence of light fields, however, the two-body potential is dominated at low densities by the dipole-dipole interaction between ground and excited atoms. Neglecting the short-range interactions, subsequently approximating the dipole-dipole potential by a “contact” potential, and performing the Hartree approximation yields an effective single-particle nonlinear Schrödinger equation which forms the basis of nonlinear atom optics [4]. Although this equation has the same structure as the Gross-Pitaevskii equation, it describes substantially different physics.

However, since spontaneous emission cannot be ignored when excited atomic levels are involved (and in addition is the cause of the dipole-dipole interaction) the nonlinear Schrödinger equation does not give an appropriate description of the system. Rather, it must be replaced by a master equation approach. This last equation results from the common procedure of eliminating the degrees of freedom of the continuum of modes of the electromagnetic field in the Born-Markoff approximation. Invoking in addition the Hartree-Fock factorization Ansatz results in the effective single particle nonlinear master equation [16,14]

$$\begin{array}{l} \frac{\partial \rho (a;b)}{\partial t}=-\frac{i}{\hbar }\langle a\left|\left[H,\rho \right]\right|b\rangle +\langle a\left|L_{nc}\rho \right|b\rangle \\ -\frac{i}{\hbar }\int d\{i\}[\langle a:1\left|\left(V+V_{c}\right)\right|2:3\rangle (\rho \left(2;b\right)\rho \left(3;1\right)\\ +\rho \left(3;b\right)\rho \left(2;1\right))\\ -\left(\rho \left(a;1\right)\rho \left(3;2\right)\right)+\rho \left(a;2\right)\rho \left(3;1\right))\\ \;\langle 1:2\left|(V+\right|V_{c})|b:3\rangle ]. \end{array}$$ (1)

Here *r* (*a*;*b*) are the matrix elements of the single particle density operator *ρ*, the “integration over numbers” such as *d*{*i*} means a summation over a complete set of quantum numbers for the single particle system, *H* is the single atom Hamiltonian, ***L****_nc_ρ* is the usual single atom spontaneous emission term, the two-body potential *V* describes the dipole-dipole interaction, and *V_c_* is an imaginary potential which results from the elimination of the modes of the thermal electromagnetic field in the Born-Markoff approximation.

We specialize to the case of a system of two-level atoms with lower electronic level |*g*〉 and upper electronic level |*e*〉. The single atom Hamiltonian then takes the form

$$H=\frac{p^{2}}{2m}+\hbar \omega_{0}\sigma_{+}\sigma_{-},$$ (2)

where ***p*** is the atomic center of mass momentum, *m* is the single particle mass, *ω*_0_ = *k*_0_*c* is the atomic transition frequency and *σ*_±_ are the usual pseudospin raising and lowering operators. The dipole-dipole potential *V* takes the explicit form [17–19]

$$\begin{array}{l} V=\frac{\hbar \Gamma }{2}\left(N-1\right)\{y_{0}(k_{0}|r_{12}|)-\frac{1}{2}\{1-3(e(r_{12})\cdot (e(d){)}^{2}\}\\ \;\times y_{2}(k_{0}|r_{12}|)\}(\sigma^{+}⊗\sigma^{-}+\sigma^{-}⊗\sigma^{+}) \end{array}$$ (3)

and the imaginary potential *V_c_* is

$$\begin{array}{l} V_{c}=i\frac{\hbar \Gamma }{2}\left(N-1\right)\{j_{0}(k_{0}|r_{12}|)-\frac{1}{2}\{1-3(e(r_{12})\cdot (e(d){)}^{2}\}\\ \;\times j_{2}(k_{0}|r_{12}|)\}\{\sigma^{-}⊗\sigma^{+}-\sigma^{+}⊗\sigma^{-}\}. \end{array}$$ (4)

In this expression, *j_i_*, *y_i_* are modified spherical Bessel functions of the first and second kind respectively [20], *N* notes the number of atoms in the sample, $\Gamma ={\left|d\right|}^{2}k_{0}^{3}/3\hbar$ is the spontaneous emission rate and ***d*** the dipole matrix element of the atomic transition. The relative position of the atoms is denoted ***r***_12_ and ***e*** (***x***) is a unit vector in the direction of the vector ***x***. In the following, we approximate these potentials by a contact potential of the form

$$\begin{array}{c} V+V_{c}=\frac{V_{0}}{2k_{0}^{3}}\delta (r_{12})(\sigma^{+}⊗\sigma^{-}+\sigma^{-}⊗\sigma^{+})\\ +\frac{iV_{c0}}{2k_{0}^{3}}\delta (r_{12})(\sigma^{-}⊗\sigma^{+}+\sigma^{+}⊗\sigma^{-}), \end{array}$$ (5)

an approximation adequate for ultra-cold atoms when the thermal de Broglie wavelength of the atoms is much larger than an optical wavelength.

## 3. Coherent Regime

Having established our model, we briefly review matter waves phase conjugation in the coherent regime. In this case, the dynamics of the system is described by the Hartree nonlinear Schrödinger equation [4]

$$\begin{array}{l} i\hbar \frac{\partial \phi (a)}{\partial t}=\int d2\langle a\left|H\right|2\rangle \phi (2)\\ +\int d1d2d3\langle a,1\left|V\right|2,3\rangle \phi *(1)\phi (2)\phi (3). \end{array}$$ (6)

Note that in this equation, we have dropped the imaginary potential *V_c_*. While both *V* and *V_c_* find their physical origin in spontaneous emission, *V* conserves the purity of the state, while in general *V_c_* does not conserve *Tr* (*ρ*^2^) [14]. As such, it should really be considered a dissipative term, and consistently, be neglected in the coherent regime.

In complete analogy with conventional nonlinear optics [21–23] we proceed by introducing slowly varying forward ***F*** (***r***, *t*) ≡ (*F_e_*(***r***, *t*), *F_g_*(***r***, *t*)) and backward ***B***(***r***, *t*) ≡ (*B_e_*(***r***, *t*), *B_g_*(***r***, *t*)) propagating components of the effective single particle wave function *ϕ* (***r***, *t*)

$$\phi (r,t)=F(r,t)e^{i(kz-\omega t)}+B(r,t)e^{-i(kz+\omega t)},$$ (7)

with |∇^2^ *F_ℓ_* (***r***, *t*) | << *k* |∇*F_ℓ_* (***r***, *t*) | << *k* ^2^|*F_ℓ_* (***r***, *t*) | and |∇^2^ ***B****_ℓ_* (***r***, *t*) | << *k* |∇***B****_ℓ_* (***r***, *t*) | << *k* ^2^|***B****_ℓ_* (***r***, *t*)|, where *ℓ* = {*e*, *g*} labels the electronic state of the atoms. Here, we assume that the main direction of propagation of the atoms is along the *z*-axis, see Fig. 1. When substituted into the nonlinear Schrödinger equation, Eq. (6) with the explicit form of the dipole-dipole potential *V*, this decomposition yields a system of four coupled paraxial wave equations

$$\begin{array}{l} \left(\frac{\partial }{\partial t}+2k\frac{\partial }{\partial z}\right)\;F_{e}=i\nabla_{⊥}^{2}F_{e}\\ \;-iV_{0}({\left|F_{g}\right|}^{2}F_{e}+{\left|B_{g}\right|}^{2}F_{e}+F_{g}B_{g}^{*}B_{e})\\ \left(\frac{\partial }{\partial t}+2k\frac{\partial }{\partial z}\right)\;B_{e}=i\nabla_{⊥}^{2}B_{e}\\ \;-iV_{0}({\left|F_{g}\right|}^{2}B_{e}+{\left|B_{g}\right|}^{2}B_{e}+B_{g}F_{g}^{*}F_{e})\\ \left(\frac{\partial }{\partial t}+2k\frac{\partial }{\partial z}\right)\;F_{g}=i\nabla_{⊥}^{2}F_{g}\\ \;-iV_{0}({\left|F_{e}\right|}^{2}F_{g}+{\left|B_{e}\right|}^{2}F_{g}+F_{e}B_{e}^{*}B_{g})\\ \left(\frac{\partial }{\partial t}+2k\frac{\partial }{\partial z}\right)\;B_{e}=i\nabla_{⊥}^{2}B_{g}\\ \;-iV_{0}({\left|F_{e}\right|}^{2}B_{g}+{\left|B_{e}\right|}^{2}B_{g}+B_{e}F_{e}^{*}F_{g}), \end{array}$$ (8)

where we have introduced the dimensionless wave-number *k* → *k/k*_0_, time *t* → *ω*_rec_*t*, wavefunction *ϕ* (*y*, *z*, *t*) → *ϕ* (*y*, *z*, *t*)/*k*_0_ and position ***r*** → *k*_0_***r*** variables, as well as a dimensionless potential *V*_0_ → *V*_0_/*ω*_rec_. Equations (8) resemble the usual model equations for beam propagation in nonlinear optics [21], except that due to the vectorial character of the slowly varying amplitudes ***F*** (***r***, *t*) and ***B(r***, *t*) and the exchange of excitation involved in the dipole-dipole interaction, the nonlinear term only couples partial waves corresponding to different electronic states. As such, these equations are reminiscent of those involving polarization coupling in nonlinear optics, although in the latter case, terms involving self-phase modulation would also appear. Still, the analogy with nonlinear optics is sufficient in that we can readily adapt the beam propagation method common in optics to the problem at hand.

For numerical purposes, we restrict our attention to two dimensions and to the portion of the condensate confined by the planes *z* = 0 and *z* = *L* and assume that the boundary conditions are provided by the waves incident on these planes from the outside of that volume.^1^ If we assume that the Bose condensate consists of ground state atoms sufficiently far from the grating, we have

$$\begin{array}{l} F_{e}(y,0;t)≃{ℱ}_{e0}(0,t)+{ℱ}_{es}(0,t)e^{i\kappa y}\\ F_{g}(y,0;t)≃{ℱ}_{g0}(0,t)+{ℱ}_{gs}(0,t)e^{i\kappa y}\\ B_{e}(y,L;t)=0\\ B_{g}(y,L;t)={ℬ}_{g0}(L,t). \end{array}$$ (9)

Here, we have introduced a spatial Fourier decomposition of the transverse variation of the amplitudes to account for diffraction by the grating, which is assumed to leave the atoms in a superposition of their excited and ground electronic states. Hence ℱ*_e_*_0_(*z*, *t*), ℱ*_g_*_0_(*z*, *t*) and ℬ*_g_*_0_(*z*, *t*) are the amplitudes of the forward and backward propagating pump beams along the *z*-axis, while ℱ*_e_*_s_(*z*, *t*) and ℱ*_g_*_s_(*z*, *t*) are the amplitudes of the forward propagating probe waves propagating at a small angle to the *z*-axis such that *κ* << *k*.

For pump waves strong enough that the undepleted pump approximation holds, i.e., ℱ*_ℓ_*_0_(*z*, *t*) ≃ const, ℬ*_ℓ_*_0_ (*z*, *t*) ≃ const, this problem can be solved analytically [21,22]. It was shown in Ref. [13] that the intrinsic nonlinearity of the system results in the ground state probe being associated to an excited state conjugate wave propagating with transverse wave number −*κ*, and similarly, the excited probe leads to a ground state conjugate.

**Fig. 2:**
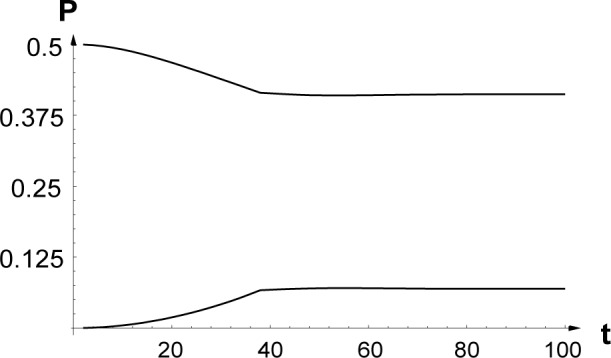
Time-dependence of the backward propagating atomic beams |ℬ*_g_*(−*κ*, 0, *t*)|^2^ — dashed line, and |ℬ*_g_*(0, 0, *t*)|^2^ — solid line, for *κ* = *k*_0_, *k* = 10*k*_0_, *L* = 40/*k*_0_, *V*_0_ = 0.4*ω_rec_*, *V_c_*_0_ = 0, *Γ* = 0, ℱ*_e_*_0_ = 0.5, ℱ*_es_* = 0.5, ℱ*_g_*_0_ = 0, ℱ*_gs_* = 0 and ℬ*_g_*_0_ = 0.7.

We performed numerical simulations of the coupled paraxial wave equations, Eq. (8) for various boundary conditions involving different fractions *η* of excited atoms in the diffracted wave at *z* = 0. As a numerical check, we verified that the atomic flux through the *z* = 0 and *z* = *L* planes is properly conserved. Fig. 2 shows an example of the build-up of the ground state phase conjugate wave of amplitude ℬ*_g_* (−*κ*, 0, *t*), and the associated depletion of the pump amplitude ℬ*_g_* (0, 0, *t*).

The dipole-dipole potential *V* responsible for the non-linearity is switched on at time *t* = 0 and the atoms are assumed to be left in their excited state after being diffracted by the grating. In that case, the conjugate beam builds up only in the electronic ground state as expected from the analytical solution in the undepleted regime [13]. The partial Schrödinger waves first undergo a transient, with discontinuities in their slope after the beams cross the condensate an integer number of times.^2^ The kicks become less and less pronounced as a new steady state corresponding to *V* ≠ 0 is established.

**Fig. 3:**
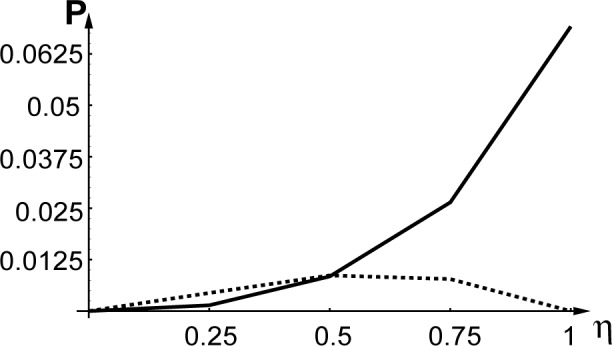
Strength of the conjugate beams |ℬ*_e_*(−*κ*, 0, *t*)|^2^ — dashed line, and |ℬ*_g_*(−*κ*, 0, *t*)|^2^ — solid line, in steady state, as a function of the fraction *η* of excited atoms in the reflected beam. *κ* = *k*_0_, *k* = 10*k*_0_, *L* = 40/*k*_0_, *V*_0_ = 0.4*ω_rec_*, *V_c_*_0_ = 0 and *Γ* = 0.

Figure 3 shows how the fraction of excited state atoms in the reflected beam influences the intensity of the conjugate beams. As follows both from analytical considerations [13] and from our numerical simulations, no conjugate beams are produced if the atoms exit the grating in their ground state. This is simply because in that case, there is no spontaneous emission, and no dipole-dipole interaction between the atoms. Note the asymmetry between the excited and ground state atomic conjugation, which results from the asymmetry in the pump beams, the condensate being assumed to consist of ground state atoms. Of course, the results for large fractions of excited atoms should be regarded with caution, due to the neglect of the incoherent effects of spontaneous emission in these results. This is the object of the next section.

## 4. Spontaneous Emission

### 4.1 Master equation approach

In order to account for the effects of spontaneous emission, the Hartree nonlinear Schrödinger equation, Eq. (6) must be replaced by the nonlinear master equation, Eq. (1), as already mentioned. In analogy with the coherent case, we now expand the effective single particle density matrix element *ρ_ℓℓ_*_′_ (***r***, ***r***′, *t*) as

$$\begin{array}{l} \rho_{\ell \ell^{\prime }}(r,r^{\prime },t)=\rho_{\ell \ell^{\prime }}^{ff}(r,r^{\prime },t)e^{ik(z-z^{\prime })}+\rho_{\ell \ell^{\prime }}^{bb}(r,r^{\prime },t)e^{-ik(z-z^{\prime })}\\ +\rho_{\ell \ell^{\prime }}^{fb}(r,r^{\prime },t)e^{ik(z+z^{\prime })}+\rho_{\ell \ell^{\prime }}^{bf}(r,r^{\prime },t)e^{-ik(z+z^{\prime })}. \end{array}$$ (10)

This decomposition yields 16 coupled paraxial-like partial differential equations of the spatial variables (*y*, *z*), (*y*′, *z*′) and *t*. Their explicit form is given in Appendix A. These equations, being first-order in *z* and *z*′, require boundary conditions on the four sides of the square *z*, *z*′ **ϵ** [0, *L*]. However, the boundary conditions Eq. (9) on the wave functions in the coherent case yield boundary conditions for the density matrix at the corners of this square only. The additional boundary equations required for the sides of the square can be derived from the Hartree nonlinear Schrödinger equation with the complex potential *V* + *V_c_*, as shown in Appendix A.

We have numerically solved the Hartree-Fock nonlinear master equation in two dimensions for various spontaneous decay rates and fractions *η* of excited atoms at the exit of the beam splitter. As we are interested in particular transverse directions of the wave propagation we introduce a spatial Fourier transform of the atomic density operator in the transverse dimension

$$\begin{array}{l} {♇}_{\ell \ell^{\prime }}^{\alpha \beta }(k_{y},z;{k^{\prime }}_{y},z^{\prime },t)=\\ \;\int dk_{y}d{k^{\prime }}_{y}\rho_{\ell \ell^{\prime }}^{\alpha \beta }(y,z;y^{\prime },z^{\prime },t)e^{ik_{y}y}e^{i{k^{\prime }}_{y}y^{\prime }}, \end{array}$$ (11)

where *α* and *β* stand for the forward and backward propagating wave.^3^

**Fig. 4:**
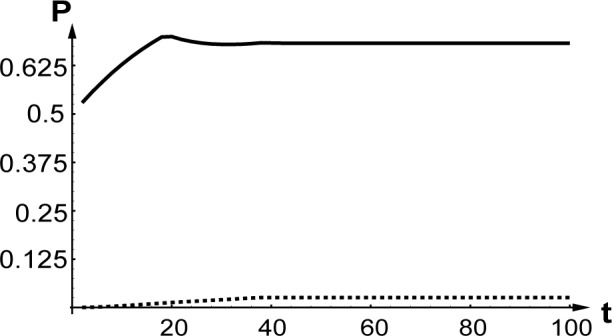
Master equation simulations in the incoherent regime. Time-dependence of the backward propagating atomic beams ${♇}_{gg}^{bb}(0,0;0,0,t)$ — solid line, ${♇}_{gg}^{bb}(-\kappa ,0;-\kappa ,0;t)$ — dashed line, for *κ* = *k*_0_, *k* = 10*k*_0_, *L* = 40/*k*_0_, *V*_0_ = 0.2*ω_rec_*, *V_c_*_0_ = 0.1*ω_rec_*, *Γ* = 0.5*ω_rec_*, *k*_spont_ = *k*_0_, ℱ*_e_*_0_ = 0.5, ℱ*_es_* = 0.5, ℱ*_g_*_0_ = 0, ℱ*_gs_* = 0 and ℱ*_g_*_0_ =0.7.

**Fig. 5:**
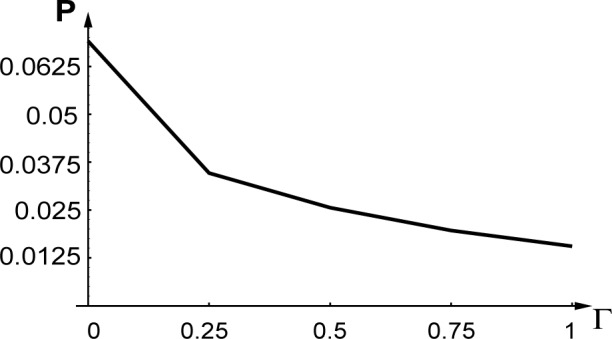
Influence of *Γ* on the strength of the conjugate beam ${♇}_{gg}^{bb}(-\kappa ,0;-\kappa ,0;t)$ in steady state *κ* = *k*_0_, *k* = 10*k*_0_, *L* = 40/*k*_0_, *V*_0_ = 0.2*ω_rec_*, *V_c_*_0_ = 0. 1*ω_rec_*, *k*_spont_ = *k*_0_, ℱ*_e_*_0_ = 0.5, ℱ*_es_* = 0.5, ℱ*_g_*_0_ = 0, ℱ*_gs_* = 0 and ℱ*_g_*_0_ = 0.7.

Figure 4 shows the probabilities ${♇}_{gg}^{bb}(-\kappa ,0;-\kappa ,0,t)$ and ${♇}_{gg}^{bb}(0,0;0,0,t)$ as a function of time, for the case where the atoms exit the diffraction grating in their excited state. As such, it is the same as Fig. 2, except that now the spontaneous emission rate has been set equal to *Γ* = 0.5 and the imaginary potential is *V_c_* = 0.1, in recoil units. Clearly, the amount of phase conjugation is substantially reduced as compared to the coherent situation. This is further illustrated in Fig. 5, which shows the steady state phase conjugate probability as a function of the spontaneous decay rate.

Already for a decay rate equal to the recoil frequency, phase conjugation has all but disappeared.^4^

### 4.2 Hartree Monte Carlo Simulations

The numerical solution of the nonlinear master equation leads to substantial computer memory requirements. In a recent paper [24], we have proposed an alternative approach to its brute force solution, based on the average over “quantum trajectories” resulting from the solution of a Hartree nonlinear Schrödinger equation, interrupted by quantum jumps. The validity of this approach, which is inspired by the now well-established Monte Carlo wave functions technique [25–27], can however only be proven provided that the Hartree approximation is exact, which holds strictly only for condensates. In addition, the two-body contribution to dissipation must be of the form of a complex potential, which is the case in the present situation. A direct comparison of the Hartree Monte Carlo and master equation results should therefore give us not only an indicator of the practical validity of the former method, but also a sense of how spontaneous emission destroys the condensate.

In order to implement this approach, we generalize the Hartree nonlinear Schrödinger equation, Eq. (6) to include the imaginary potential *V_c_*, so that it becomes

$$\begin{array}{l} \;i\hbar \frac{\partial \phi (a)}{\partial t}=\int d2\langle a\left|H\right|2\rangle \phi (2)\\ +\int d1d2d3\langle a,1\left|V+V_{c}\right|2,3\rangle \phi *(1)\phi (2)\phi (3). \end{array}$$ (12)

Expressing furthermore the *linear* Liouvillian ***L****_nc_* in Eq. (1) as the Lindblad form

$$\begin{array}{c} L_{nc}\rho (t)=-\frac{1}{2}\sum_{m}\left(C_{m}^{†}C_{m}\rho (t)+\rho (t)C_{m}^{†}C_{m}\right)\\ +\sum_{m}C_{m}\rho (t)C_{m}^{†} \end{array}$$ (13)

where the *C_m_*’s are system operators appearing in its coupling to the continuum of electro-magnetic field modes, the Hartree Monte Carlo algorithm proceeds as follows [24]. We first formally reexpress the nonlinear Schrödinger equation, Eq. (12) as

$$i\hbar |\phi \left(t\right)\rangle =H_{nl}[|\phi \left(t\right)\rangle ]|\phi \left(t\right)\rangle .$$ (14)

where the nonlinear “Hamiltonian” $H_{nl}[|\phi \left(t\right)\rangle ]$ *H_n_*[|*ϕ*(t)〉] is a functional of the effective single-particle state vector |*ϕ*(*t*)〉, see Eq. (12). We then decompose the Liouvillian ***L****_nc_* into a contribution *H_d_* added to *H_nl_* to yield the effective nonhermitian “Hamiltonian”

$$\begin{array}{c} H_{\mathrm{eff}}\left[|\phi \rangle \right]\equiv H_{nl}\left[|\phi \rangle \right]+H_{d}\\ \;=H_{nl}\left[|\phi \rangle \right]-\frac{i\hbar }{2}\sum_{m}C_{m}^{†}C_{m} \end{array}$$ (15)

and a *linear* “fill-up term,” see Eq. (13). As in the usual case, *H*_eff_ is responsible for the Schrödinger-like evolution of the system, while the “fill-up term” causes randomly distributed quantum jumps.

The probability δ*p* for one of these jumps to occur is given for the small time interval δ*t* by^5^

$$\begin{array}{l} \delta p=\frac{i\delta t}{\hbar }\frac{\langle \phi (t)\left|H_{d}-H_{d}^{†}\right|\phi (t)\rangle }{\langle \phi (t)|\phi (t)\rangle }\\ \;=\delta t\frac{\langle \phi (t)|\sum_{m}C_{m}^{†}C_{m}|\phi (t)\rangle }{\langle \phi (t)|\phi (t)\rangle }\equiv \sum_{m}\delta p_{m}. \end{array}$$ (16)

A jump is said to occur if a quasi-random number **ϵ**, uniformly distributed between 0 and 1, is less than δ*p*. The post-jump wave function is chosen amongst the various possible final states *C_m_*|*ϕ*(*t*)〉 according to the probability law δ*p_m_*/δ*p*, with

$$\left|\phi \left(t+\delta t\right)\rangle =C_{m}\right|\phi \left(t\right)\rangle /{\left(\delta p_{m}/\delta t\right)}^{1/2}.$$ (17)

In case no jump occurs, the state vector evolves under the influence of the nonlinear and nonhermitian effective Hamiltonian *H*_eff_, so that

$$\left|\phi^{\left(1\right)}\left(t+\delta t\right)\rangle ≃\right|\phi \left(t\right)\rangle -\frac{iH_{\mathrm{eff}}[\left|\phi \left(t\right)\rangle ]\right|\phi \left(t\right)\rangle }{\hbar }\delta t$$ (18)

and the new wave function is

$$\left|\phi \left(t+\delta t\right)\rangle =\right|\phi^{\left(1\right)}\left(t+\delta t\right)\rangle /\sqrt{1-\delta p}.$$ (19)

**Fig. 6:**
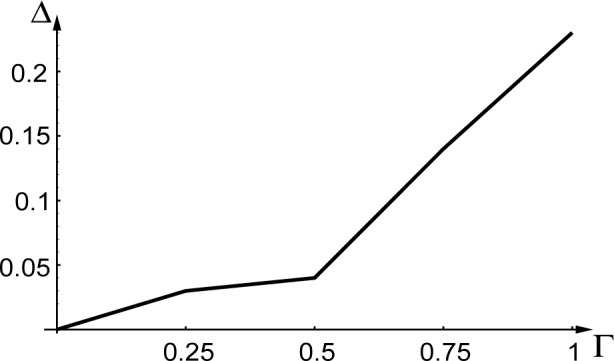
Comparison of the Monte Carlo and master equation simulations. Influence of *Γ* on *Δ*, the standard deviation between the steady state results of the Monte Carlo and master equation predictions, normalized by the Master equation result.

We performed Monte Carlo simulations within the slowly varying envelope approximation introduced in Sec. 2 and for the same beam configuration as in the preceeding example (see Appendix B). As pointed out in Ref. [24], the accuracy of the Monte Carlo simulations as compared to the Hartree-Fockmaster equation description is expected to depend on the amount of dissipation in the problem. Figure 6 shows the relative difference between the two methods as a function of the spontaneous emission rate *Γ*. These results confirm that the Hartree Monte Carlo simulations work surprisingly well, although the discrepancy between the two methods increases with *Γ*, as expected.

We attribute this surprisingly good agreement to the fact that the type of dissipation that we are considering here is however quite special, in that the only effect of the nonlinear Liouvillian ***L****_nl_* is in the form of an imaginary two-body potential *V_c_*. It corresponds to a modification of the spontaneous decay rate resulting from the interference of the spontaneous emission probability amplitudes due to the undistinguishability of the two particles. In other words, it describes a *collective* decay mechanism (albeit as a result of the Born-Markov approximation, only two-atom effects are retained). Due to this collective nature, it is reasonable to expect that this imaginary potential might not do too much violence to the Hartree ansatz.

The only other incoherent contribution to the nonlinear master equationis a *single-particle* Liouvillian ***L****_r_*. But due to the indistinguishability of the particles in the sample, it describes a decay mechanism that occurs for all *N* atoms simultaneously. As a result, it leaves all atoms in the same state, albeit now described by a product of *N* identical single-particle density operators. It appears then that for the very specific form of decay we are considering, the Hartree ansatz might not be as bad as otherwise expected, a conjecture largely confirmed by the result of Fig. 6.

## 5. Summary and Conclusions

In this paper we have discussed the possibility of observing atomic phase conjugation off Bose condensates, and to use it as a diagnostic tool to determine the spatial coherence properties and lifetime of that condensate. Since phase conjugation results from the scattering of a partial matter wave off the spatial grating produced by two other waves, it offers a very natural way to directly measure these coherence properties, and as such provides an attractive alternative to the optical methods proposed in the past.

As expected, incoherent mechanisms such as spontaneous emission rapidly destroy phase conjugation, thereby demonstrating that the proposed technique should easily distinguish a condensate from an incoherent sample of the same density. We have also compared Hartree-Fock master equation results to those based on Hartree Monte Carlo wave function simulations, thereby obtaining a measure of the destruction of the condensate as a function of the rate of spontaneous emission.

We conclude by noting that in replacing the nonlocal dipole-dipole potential by a local interaction, the gratings established in the condensate are probed only locally. It will be interesting to investigate if more information can be gained by treating *V* as a nonlocal potential. In particular, one can expect that effects such as spatial diffusion in the sample should be accessible by such nonlocal sampling.

## Footnotes

1 The *x*-dimension is eliminated by expressing the wave function *ϕ_ℓ_* (***r***, *t*) as *ϕ* (*x*)*ϕ_ℓ_* (*y*, *z*, *t*) where *ϕ* (*x*) is taken to be a solution of a linear Schrödinger equation. As a result *V*_0_ is replaced by *V*_0_ ∫ d*x* |*ϕ*(*x*)|^4^/*k*_0_.

2 This is an artifact of our numerical algorithm, which starts propagating the states from the two boundaries at *z* = 0 and *z* = *L* after *V* is switched on.

3 As it should be, our numerical results agree with the coherent ones for *Γ* = *V_c_* = 0.

4 The parameters of our simulations were largely dictated by computer limitations. It is expected that phase conjugation will survive larger spontaneous emission rates if larger dipole-dipole potentials and longer propagation distances are considered.

5 Note that we keep track of the norm of the state vector explicitly, since only a partial volume of the system is considered explicitly, and thus the flux, rather than the norm, is conserved in our numerical solution.

## 6. Appendix A. Master Equation in the Slowly Varying Envelope Approximation

In analogy to the wave function description in terms of forward and backward propagating beams each of the four density matrix elements *ρ_ee_*, *ρ_gg_*, *ρ_eg_*, *ρ_ge_* can be decomposed into four parts

$$\begin{array}{l} \rho_{\ell \ell^{\prime }}\left(r,r^{\prime },t\right)=\rho_{\ell \ell^{\prime }}^{ff}\left(r,r^{\prime },t\right)e^{ik(z-z^{\prime })}+\rho_{\ell \ell^{\prime }}^{bb}\left(r,r^{\prime },t\right)e^{-ik(z-z^{\prime })}\\ \;+\rho_{\ell \ell^{\prime }}^{fb}\left(r,r^{\prime },t\right)e^{ik(z+z^{\prime })}+\rho_{\ell \ell^{\prime }}^{bf}\left(r,r^{\prime },t\right)e^{-ik(z+z^{\prime })}, \end{array}$$ (20)

where

$$\rho_{\ell \ell^{\prime }}^{fb}=F_{\ell }(r,t)B_{\ell^{\prime }}^{*}(r^{\prime },t).$$ (21)

The 16 coupled paraxial-like partial differential equations obtained from the nonlinear master equation, Eq. (1) in the slowly varying envelope approximation are of the form

$$\begin{array}{l} \left(\frac{\partial }{\partial t}+2k\left(\frac{\partial }{\partial z}+\frac{\partial }{\partial z^{\prime }}\right)\right)\rho_{ee}^{ff}\left(r,r^{\prime };t\right)=\\ \;i\left(\nabla_{⊥}^{2}-\nabla_{{⊥}^{\prime }}^{2}\right)\rho_{ee}^{ff}\left(r,r^{\prime };t\right)\\ -iV_{t0}^{*}[\left(\rho_{eg}^{ff}\left(r,r;t\right)+\rho_{eg}^{bb}\left(r,r;t\right)\right)\rho_{eg}^{ff}\left(r,r^{\prime };t\right)+\\ \;\rho_{eg}^{fb}\left(r,r;t\right)\rho_{ge}^{bf}\left(r,r^{\prime };t\right)]\\ -iV_{t0}^{*}[\left(\rho_{gg}^{ff}\left(r,r;t\right)+\rho_{gg}^{bb}\left(r,r;t\right)\right)\rho_{ee}^{ff}\left(r,r^{\prime };t\right)+\\ \;\rho_{gg}^{fb}\left(r,r;t\right)\rho_{ee}^{bf}\left(r,r^{\prime };t\right)]\\ +iV_{t0}[\left(\rho_{ge}^{ff}\left(r^{\prime },r^{\prime };t\right)+\rho_{ge}^{bb}\left(r^{\prime },r^{\prime }t\right)\right)\rho_{eg}^{ff}\left(r,r^{\prime };t\right)+\\ \;\rho_{ge}^{bf}\left(r^{\prime },r^{\prime };t\right)\rho_{eg}^{fb}\left(r,r^{\prime };t\right)]\\ +iV_{t0}[\left(\rho_{gg}^{ff}\left(r^{\prime },r^{\prime };t\right)+\rho_{gg}^{bb}\left(r^{\prime },r^{\prime };t\right)\right)\rho_{ee}^{ff}\left(r,r^{\prime };t\right)+\\ \;\rho_{gg}^{bf}\left(r^{\prime },r^{\prime };t\right)\rho_{ee}^{fb}\left(r,r^{\prime };t\right)]\\ +\langle er^{\prime }\left|L_{nc}\rho_{ee}^{ff}\left(t\right)\right|er\rangle , \end{array}$$ (22)

where *V_t_*_0_ = *V*_0_ + *V_c_*_0_. Similar equations can be obtained for all other components.

These first-order differential equations in *z* and *z*′ require boundary conditions on the four sides of the square *z*, *z*′ = [0, *L*]. However, the boundary conditions for the wave functions in the coherent case Eq. (9) yield boundary conditions for the density matrix only at the corners of the square. Ignoring spontaneous emission at the boundaries, we can express the density matrix elements as $\rho_{ee}^{ff}(y,z,y^{\prime },0,t)=F_{e}(y,z,t)F*(y^{\prime },0,t)$, etc. In this expression, *F_e_* (*y*, *z*, *t*) undergoes a Schrödinger evolution, while *F**(*y*′, 0, *t*) is a boundary value. Thus, the dynamics of the system on the sides of the square is obtained by considering a *boundary equation* for the master equation, which involves only the evolution corresponding to the change of “half” of the density matrix element.

This boundary equation can be derived from the Hartree nonlinear Schrödinger equation with the potential *V* + *V_c_*. In particular, we find for $\rho_{ee}^{ff}(r,{r^{\prime }}_{\mathrm{bound}};t)$

$$\begin{array}{l} \left(\frac{\partial }{\partial t}+2k\frac{\partial }{\partial z}\right)\rho_{ee}^{ff}\left(r,{r^{\prime }}_{\mathrm{bound}};t\right)=\\ \;i\;\nabla_{⊥}^{2}\;\rho_{ee}^{ff}\left(r,{r^{\prime }}_{\mathrm{bound}};t\right)\\ -iV_{t0}^{*}[\left(\rho_{eg}^{ff}\left(r,r;t\right)+\rho_{eg}^{bb}\left(r,r;t\right)\right)\rho_{ge}^{ff}\left(r,{r^{\prime }}_{\mathrm{bound}};t\right)+\\ \;\rho_{eg}^{fb}\left(r,r;t\right)\rho_{ge}^{bf}\left(r,{r^{\prime }}_{\mathrm{bound}};t\right)]\\ -iV_{t0}^{*}[\left(\rho_{gg}^{ff}\left(r,r;t\right)+\rho_{gg}^{bb}\left(r,r;t\right)\right)\rho_{ee}^{ff}\left(r,{r^{\prime }}_{\mathrm{bound}};t\right)+\\ \;\rho_{gg}^{fb}\left(r,r;t\right)\rho_{ee}^{bf}\left(r,{r^{\prime }}_{\mathrm{bound}};t\right)] \end{array}$$ (23)

Here ***r***′_bound_ is the position coordinate on the boundary, ***r***′_bound_ = (*x*′, *y*′, 0) for the forward propagating component and ***r***′_bound_ = (*x*′, *y*′, *L*) for the backward propagating component. Note that this situation is different from the case of confined systems, where *ρ_ℓℓ_*_′_ (*y*, *z*; *y*′, *z*′_bound_, *t*) = 0 on the boundary.

## 7. Appendix B. Explicit Expressions for the Hartree Monte Carlo Simulations

The explicit form of the incoherent Liouvillian ***L****_nc_* in Eq. (1) leads to the system operators

$$C_{\chi }^{\pm }=\sqrt{\frac{3\Gamma }{4}(1-\chi^{2})}\sigma^{\pm }e^{\pm i\frac{\omega_{0}}{c}e(k)r},$$ (24)

where 3 (1 − *χ*^2^)/4 is the dipole radiation pattern for the two-level atoms and χ = cos*θ*, *θ* being the angle between the quantization axis and the direction in which the spontaneous photon is emitted and exp $\left(\pm i\frac{\omega_{0}}{c}e(k)r\right)$ resembles atomic recoil due to the spontaneous emission event [26]. The effective Hamiltonian *H*_eff_ [|*ϕ*〉] is then

$$H_{\mathrm{eff}}\left[|\phi \rangle \right]=H_{nl}\left[|\phi \rangle \right]-\frac{i\hbar \Gamma }{2}\sigma^{+}\sigma^{-}$$ (25)

and yields the quantum jump probability

$$\begin{array}{l} \delta p=\frac{3\Gamma }{4}\delta t\frac{{\left|\phi_{e}(t)\right|}^{2}}{{\left|\phi (t)\right|}^{2}}\int_{-1}^{1}d\chi (1-\chi^{2})\\ \;\equiv \sum_{\chi }\delta p_{\chi }. \end{array}$$ (26)

Finally, the post-jump wave function turns out to be

$$|\phi (t+\delta t)\rangle =\frac{e^{-i\frac{\omega_{0}}{c}e(k)r}\sigma^{-}|\phi (t)\rangle }{\sqrt{\frac{{\left|\phi_{e}(t)\right|}^{2}}{{\left|\phi (t)\right|}^{2}}}}$$ (27)

i.e., *ϕ_e_* (*t* + δ*t*) = 0 and

$$\phi_{g}(t+\delta t)=e^{-i\frac{\omega_{0}}{c}e(k)r}\sigma^{-}|\phi_{e}(t)\rangle /\sqrt{{\left|\phi_{e}(t)\right|}^{2}/{\left|\phi (t)\right|}^{2}}.$$

In case of decay the wave functions are given by Eq. (19).

These simulations are carried out within the slowly varying envelope approximation introduced in Sec. 2. The expressions for the slowly varying amplitudes *B_e_*(***r***,*t*+δ*t*),*B_g_* (***r***,*t*+δ*t*),*F_e_* (***r***,*t*+δ*t*) and *F_g_* (***r***,*t*+δ*t*) can be found easily from the Eqs. (17) and (19).
